# Prognostic Significance of Hypoxia-Inducible Factor Expression in Renal Cell Carcinoma: A PRISMA-compliant Systematic Review and Meta-Analysis

**DOI:** 10.1097/MD.0000000000001646

**Published:** 2015-09-25

**Authors:** Yang Fan, Hongzhao Li, Xin Ma, Yu Gao, Luyao Chen, Xintao Li, Xu Bao, Qingshan Du, Yu Zhang, Xu Zhang

**Affiliations:** From the State Key Laboratory of Kidney Diseases, Department of Urology, Military Postgraduate Medical College, Chinese People's Liberation Army General Hospital, Beijing, People's Republic of China (YF, HL, XM, LC, XL, QD, YZ, XZ); and Medical School, Nankai University, Tianjin, People's Republic of China (XB).

## Abstract

The prognostic value of hypoxia-inducible factor (HIF) in renal cell carcinoma (RCC) has been evaluated in a large number of studies, but the reports were inconsistent and remained inconclusive. Therefore, we conducted a systematic review and meta-analysis to clarify the significance of HIF expression in RCC prognosis.

PubMed, Embase, Web of Science, Cochrane Library, EBSCO, Cumulative Index to Nursing and Allied Health Literature (CINAHL), and Biological Abstracts were searched for eligible studies. Hazard ratio (HR) data for overall survival (OS), cancer-specific survival (CSS), and progression-free survival (PFS) with 95% confidence interval (CI) related to the expression status of HIF-1α or HIF-2α detected by immunohistochemistry were all extracted. Data were combined using a random- or fixed-effects model based on the corresponding inter-study heterogeneity. Subgroup analyses were also performed.

A total of 14 studies composed of 1258 patients for HIF-1α evaluation and 619 patients for HIF-2α evaluation were included for further analysis. When initially analyzed as a whole, the HIF-1α expression was not significantly correlated with OS (HR 1.637, 95% CI 0.898–2.985, *P* = 0.108), CSS (HR 1.110, 95% CI 0.595–2.069, *P* = 0.744), and PFS (HR 1.113, 95% CI 0.675–1.836, *P* = 0.674). Similarly, HIF-2α expression was not significantly correlated with CSS (HR 1.597, 95% CI 0.667–3.824, *P* = 0.293) and PFS (HR 0.847, 95% CI 0.566–1.266, *P* = 0.417). However, subgroup analyses concerning subcellular localization of HIFs revealed that the high nuclear expression of HIF-1α was significantly associated with poor OS (HR 2.014, 95% CI 1.206–3.363, *P* = 0.007) and the high cytoplasmic expression of HIF -2α was significantly associated with poor CSS (HR 2.356, 95% CI 1.629–3.407, *P* = 0.000).

The increased nuclear expression of HIF-1α and cytoplasmic expression of HIF-2α indicate unfavorable prognosis in RCC patients, which may serve as biomarkers for disease management.

## INTRODUCTION

Renal cell carcinoma (RCC), which accounts for 2% to 3% of all adult malignancies, is one of the most prevalent urologic cancers and the second leading cause of death among its cancer type.^1^ RCC is highly aggressive; ∼30% of RCC patients present metastasis at initial diagnosis, and another 20% to 30% of RCC patients with clinically localized disease eventually develop metastasis even after curative nephrectomy.^2,3^ Although surgery remains the gold standard among treatment strategies for localized RCC, this method provides limited benefits to RCC patients with locally advanced or metastatic disease; in this regard, early systematic therapy is required.^4^ Considering that the current surveillance of RCC mostly relies on imaging tests,^4^ identifying novel biomarkers to stratify patients with poor prognosis in the early stage of RCC is significantly needed.

Given that clear-cell RCC (ccRCC) represents ∼ 80% of RCC subtypes^5^ and loss of von Hippel–Lindau (VHL) tumor suppressor gene is found in the majority (75–85%) of ccRCC,^6^ VHL may play a central role in RCC biology. In the absence of a functional VHL protein, VHL-associated proteolysis of hypoxia-inducible factor (HIF) occurring in normoxia is lost. This behavior leads to an accumulation of HIF-1α and HIF-2α, as well as subsequent transcription of HIF target genes involved in angiogenesis, such as vascular endothelial growth factor (VEGF) and platelet-derived growth factor (PDGF).^7^ Although HIF-1α and HIF-2α exhibit 48% amino acid sequence identity and similar protein structures, they contain distinct target genes and regulatory mechanisms.^8,9^ With recent advancement in the understanding of molecular basis of RCC tumorigenesis and metastasis, many studies concerning HIF-1α and HIF-2α were conducted in terms of outcome prediction and potential therapeutic targets. Several studies such as Klatte et al^10^ and Minardi et al^11^ directly implicated that overexpression of HIF-1α was a critical factor in RCC development, which was associated with poor prognosis. However, Biswas et al^12^ reported that HIF-2α was more tumorigenic in RCC and others even implicated HIF-1α as a tumor suppressor gene.^13,14^ HIF was considered an unfavorable prognostic marker in other types of tumors such as colorectal cancer^15^ and gynecological cancer^16^ using meta-analysis, but its prognosis remained inconclusive in RCC patients. Hence, we conducted a systematic review and meta-analysis of eligible studies to quantitatively evaluate the prognostic values and explore the exact roles of different HIF isoforms in RCC.

## MATERIALS AND METHODS

### Search Strategy

This meta-analysis was conducted following the guidelines of Preferred Reporting Items for Systematic Reviews and Meta-Analyses (PRISMA),^17^ which is available in the supplementary materials (PRISMA Checklist). A literature search was performed until August 15, 2015, in PubMed, Embase, Web of Science, Cochrane Library, EBSCO, Cumulative Index to Nursing and Allied Health Literature (CINAHL), and Biological Abstracts by using the following terms with different combinations: (“HIF” or “hypoxia-inducible factor 1” or “HIF-1α” or “endothelial PAS domain-containing protein 1” or “hypoxia-inducible factor 2” or “EPAS1” or “HIF-2α”) and (“carcinoma” or “neoplasm” or “tumor” or “cancer” or “malignancy”) and (“kidney” or “renal”) and (“survival” or “prognosis”). A manual search through reference lists of relevant literature was additionally performed. The approval by an institutional review board is not required because this study was based on published studies.

### Criteria for Inclusion and Exclusion

Inclusion criteria were as follows: (1) HIF expression (HIF-1α or HIF-2α) should be evaluated by immunohistochemistry (IHC) in RCC tissues; (2) the association between the HIF expression and prognosis of RCC should be described; and (3) study design should be clearly defined (observational study, including retrospective or prospective). Studies were excluded if they met the following criteria: (1) not written in English; (2) reviews, abstracts, case reports, meeting records, animal, or cell line studies; (3) not providing sufficient data for estimating hazard ratios (HRs) and their 95% confidence intervals (CI) of HIF expression on the prognosis of overall survival (OS), cancer-specific survival (CSS), or progression-free survival (PFS). For overlapping articles, we only retrieved the most recent and most complete studies for further analyses.

### Data Extraction and Quality Assessment

Data from all the studies that met the inclusion criteria were extracted independently by 2 investigators (Y Fan and H Li), and any discrepancy was resolved by consulting a third investigator (X Ma). The basic characteristics of each study were recorded as follows: first author's last name, year of publication, country of origin, study design, pathological type, number of patients, patients’ age, HIF isoforms and detecting method, source and dilution of antibodies, location of protein expression, cutoff value, adjuvant therapy, follow-up period, and survival outcomes (Table 1). The primary survival outcomes associated with HIF expression were focused on OS, CSS, and PFS. HRs with corresponding 95% CIs for each outcome were also extracted. If the HR and 95% CI were not directly available, the values can be estimated as reported by Tierney et al.^18^

**TABLE 1:**
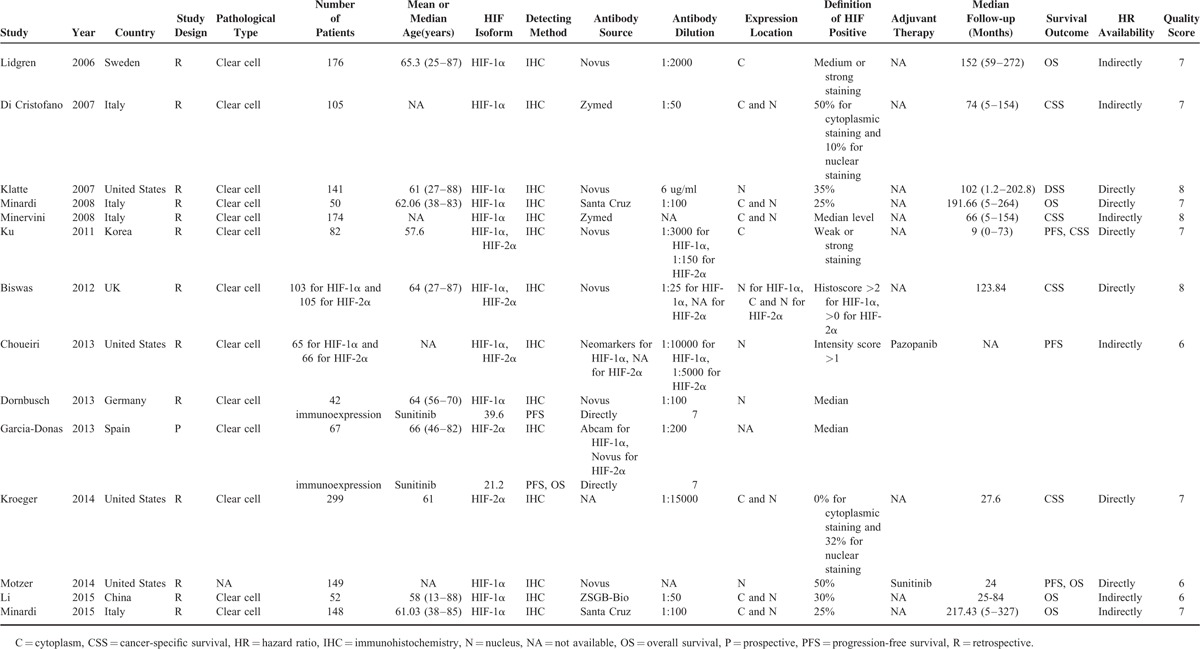
Characteristics of Eligible Studies in Meta-Analysis

The Newcastle–Ottawa scale was used to assess the quality of the included studies.^19^ Eight methodology items in each study can be assessed with a total score of 0 to 9. Studies with scores of ≥6 or more were graded as high quality. The total scores of each eligible study for this meta-analysis are listed in Table 1 and Supplementary Table 1.

### Statistical Analysis

For the pooled analysis of the association between HIF expression and prognosis, HR and 95% CI concerning OS, CSS, and PFS were used. An observed HR > 1 implied unfavorable prognosis for the group of high HIF expression. A heterogeneity test of pooled HRs was conducted using Cochran's Q test and Higgins I-squared statistic. An *I*^2^ value >50% indicated significant heterogeneity among studies. In this case, either a random- (*I*^2^ > 50%) or fixed-effects (*I*^2^ ≤ 50%) model was used. HIF-1α and HIF-2α were analyzed independently, and subgroup analyses were conducted in terms of expression location (nuclear or cytoplasmic), targeted therapy (received or not received), and HR availability (directly or indirectly). To investigate the heterogeneous studies, a sensitivity analysis was conducted to evaluate the influence of individual studies on the stability of pooled results. Publication bias was visually assessed by funnel plot and statistically evaluated by Begg's test and Egger's test. All statistical analyses were performed using Stata 12.0 software (StatCorp, College Station, TX), and a *P*-value < 0.05 was considered significant.

## RESULTS

### Eligible Studies

**FIGURE 1:**
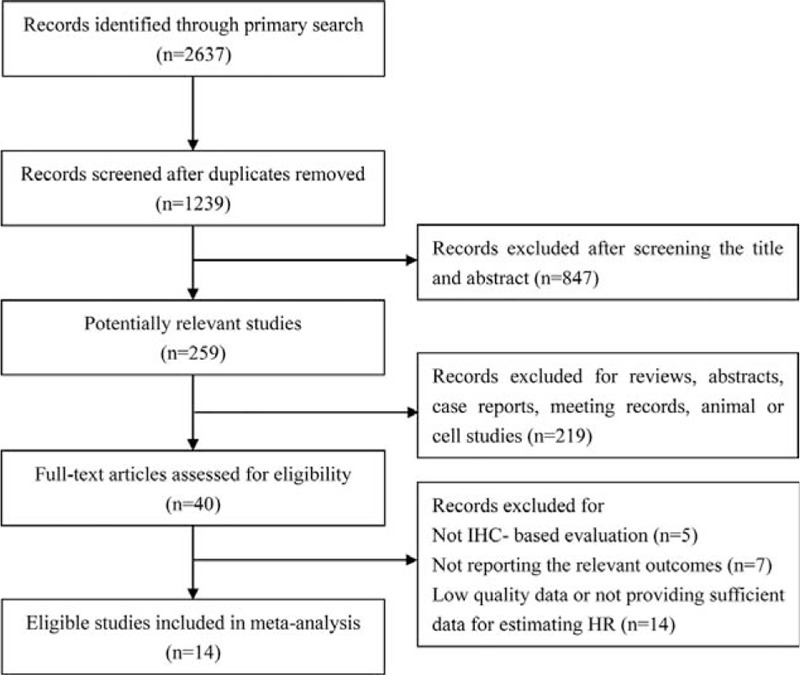
Flowchart of study selection. IHC = immunohistochemistry, HR = hazard ratio.

A total of 2637 records were retrieved from the primary literature search (through electronic databases and reference lists of relevant literature), and 1398 records were excluded for duplication. After screening the titles and abstracts, 40 articles were assessed for full-text review. To avoid the heterogeneity caused by the detection method, only the studies with IHC-based evaluation were included, and articles with no relevant outcome or insufficient data to estimate HR were further excluded. Finally, a total of 14 eligible studies composed of 1258 patients for HIF-1α evaluation and 619 patients for HIF-2α evaluation were included in this meta-analysis.^10–12,20–30^ A flowchart of the study selection process is shown in Figure 1, and the characteristics of eligible studies are presented in Table 1.

### Effects of Different HIF Isoform Expression on Survival Outcomes of RCC and Subgroup Analyses

Given that HIF-1α and HIF-2α exhibit distinct target genes and may exert different effects on RCC patients, we performed a meta-analysis of different HIF isoforms independently. The primary outcomes considered for pooled HRs were OS, CSS, and PFS. We did not evaluate the prognostic value of HIF-2α on OS because only 1 study was available in this concern.

**FIGURE 2:**
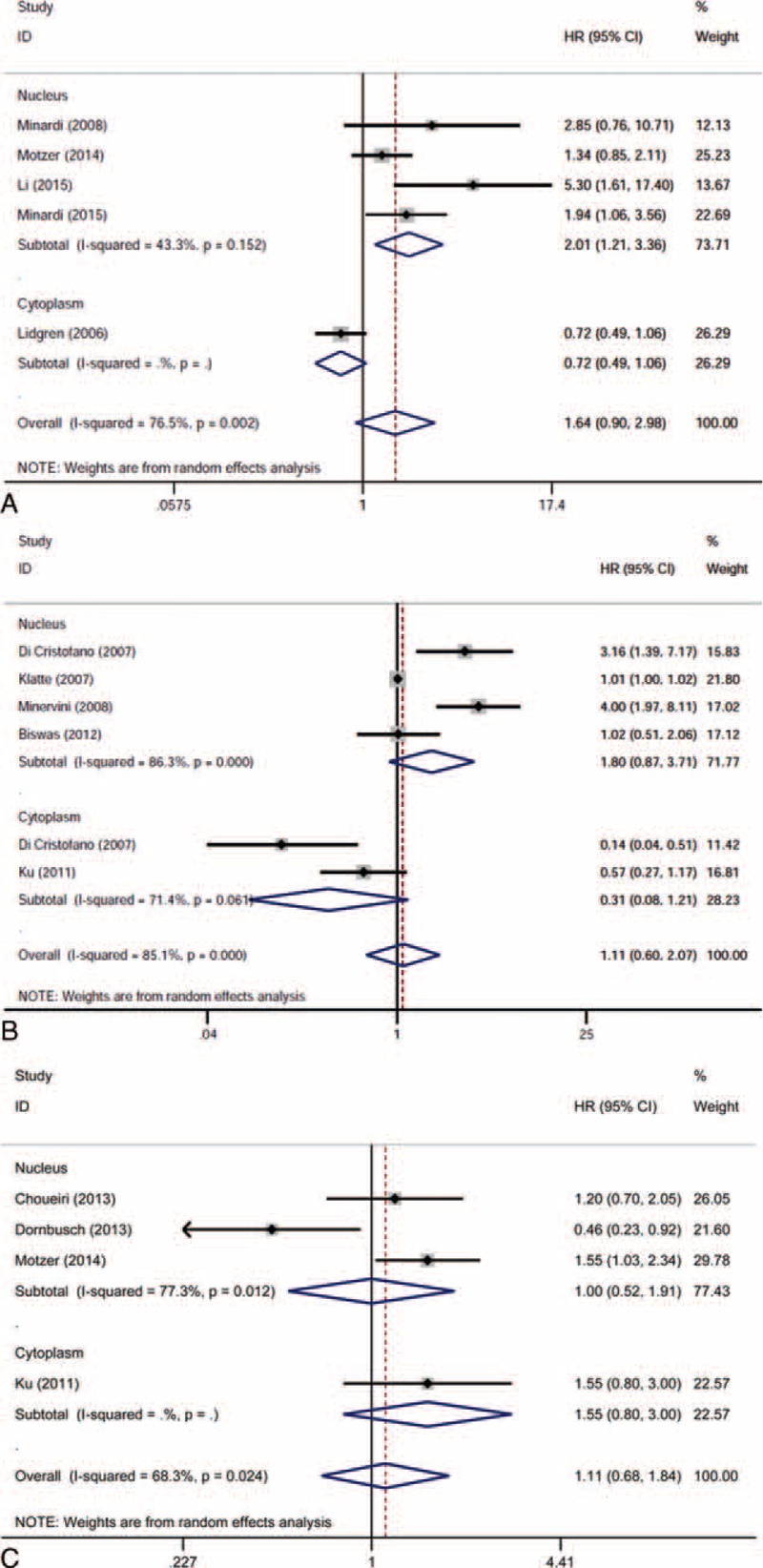
Forest plots of HR for the association of HIF-1α expression and survival outcomes of RCC patients and subgroup analysis in terms of different subcellular localization of HIF-1α expression: (A) effect of HIF-1α overexpression on OS; (B) effect of HIF-1α overexpression on CSS; (C) effect of HIF-1α overexpression on PFS. CI = confidence interval, CSS = cancer-specific survival, HIF = hypoxia-inducible factor, HR = hazard ratio, OS = overall survival, PFS = progression-free survival, HR>1 implied unfavorable prognosis, RCC = renal cell carcinoma.

**FIGURE 3:**
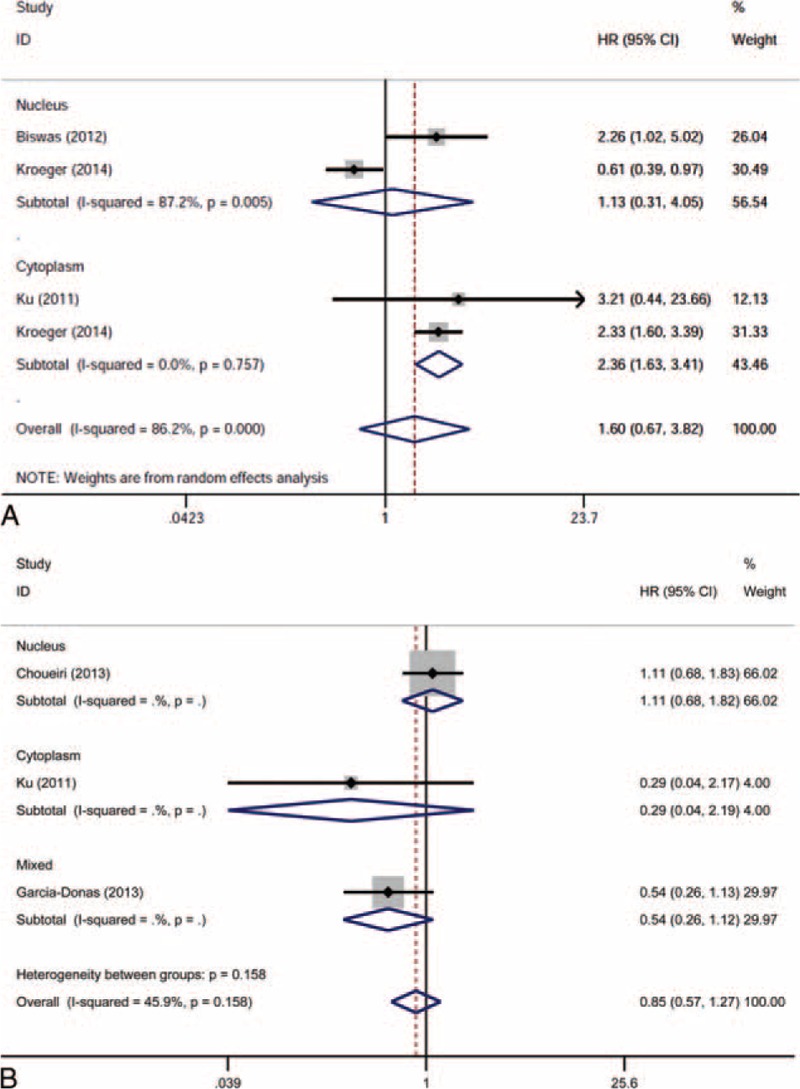
Forest plots of HR for the association of HIF-2α expression and survival outcomes of RCC patients and subgroup analysis in terms of different subcellular localization of HIF-2α expression: (A) effect of HIF-2α overexpression on CSS; (B) effect of HIF-2α overexpression on PFS. CI = confidence interval, CSS = cancer-specific survival, HIF = hypoxia-inducible factor, HR = hazard ratio, PFS = progression-free survival, HR>1 implied unfavorable prognosis, RCC = renal cell carcinoma.

The effects of different HIF isoform expressions on RCC prognosis were initially analyzed as a whole. The HIF-1α expression was not significantly correlated with OS (HR 1.637, 95% CI 0.898–2.985, *P* = 0.108; random-effects model, *I*^2^ = 76.5%, *P* = 0.002; Figure 2A), CSS (HR 1.110, 95% CI 0.595–2.069, *P* = 0.744; random-effects model, *I*^2^ = 85.1%, *P* = 0.000; Figure 2B), and PFS (HR 1.113, 95% CI 0.675–1.836, *P* = 0.674; random-effects model, *I*^2^ = 68.3%, *P* = 0.024; Figure 2C). Similarly, the HIF-2α expression was not significantly correlated with CSS (HR 1.597, 95% CI 0.667–3.824, *P* = 0.293; random-effects model, *I*^2^ = 86.2%, *P* = 0.000; Figure 3A) and PFS (HR 0.847, 95% CI 0.566–1.266, *P* = 0.417; fixed-effects model, *I*^2^ = 45.9%, *P* = 0.158; Figure 3B).

The overall insignificant results and inter-study heterogeneity led us to explore the detailed information in subgroup analyses. We first stratified the studies in each analysis group as nuclear expression and cytoplasmic expression subgroups. Interestingly, a high nuclear expression of HIF-1α was revealed to be significantly associated with poor OS (HR 2.014, 95% CI 1.206–3.363, *P* = 0.007; random-effect model, *I*^2^ = 43.3%, *P* = 0.152; Figure 2A), whereas a high cytoplasmic expression of HIF-2α was significantly associated with poor CSS (HR 2.356, 95% CI 1.629–3.407, *P* = 0.000; random-effects model, *I*^2^ = 0.0%, *P* = 0.757; Figure 3A). For the absence of obvious heterogeneity in the subgroup results above, a fixed-effects model was applied for analysis again, and the pooled HRs were 1.760 (95% CI 1.257–2.464, *P* = 0.001) and 2.356 (95% CI 1.629–3.407, *P* = 0.000) for the HIF-1α nuclear expression on OS and HIF-2α cytoplasmic expression on CSS, respectively. Further subgroup analyses were performed in terms of targeted therapy (received or not received) and HR availability (directly or indirectly), and the association of HIF expression and survival outcomes did not reach significance for the stratification (Tables 2 and 3).

**TABLE 2:**
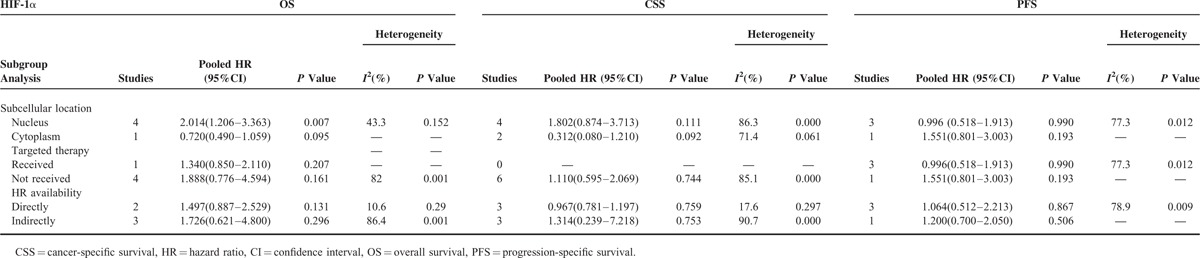
Subgroup Analysis of Pooled HR for RCC Patients With HIF-1α Overexpression

**TABLE 3:**
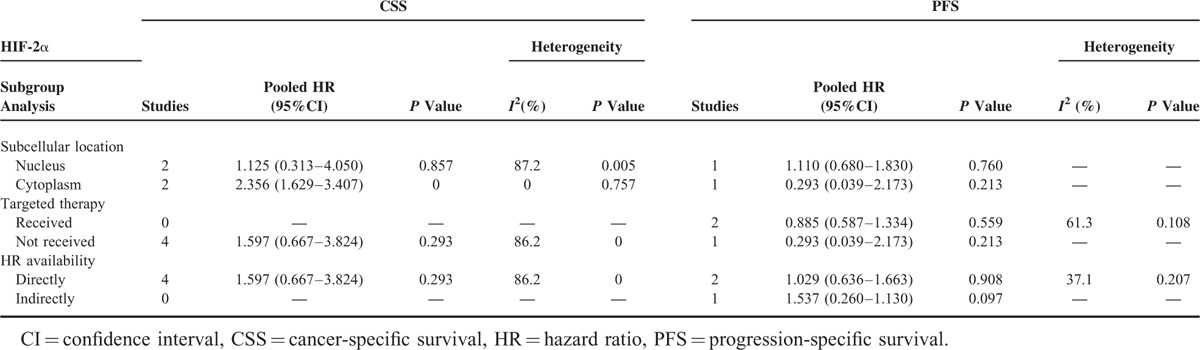
Subgroup Analysis of Pooled HR for RCC Patients With HIF-2α Overexpression

Sensitivity analysis indicated that the pooled HR was not significantly influenced if any single study was omitted in subgroup analysis for the effect of HIF-1α nuclear expression on OS (Table 4). Given that only 2 studies were included in subgroup analysis for the effect of HIF-2α cytoplasmic expression on CSS and inter-study heterogeneity was not detected (*I*^2^ = 0.0%, *P* = 0.757), sensitivity analysis was not conducted in this category.

**TABLE 4:**
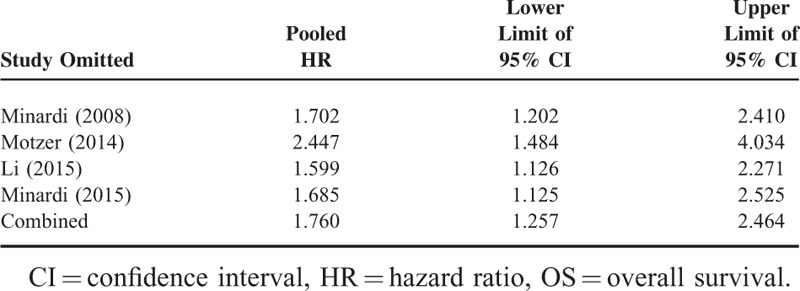
Pooled HR (95% CI) of Sensitivity Analysis for Effect of HIF-1α Nuclear Expression on OS

### Publication Bias

**FIGURE 4:**
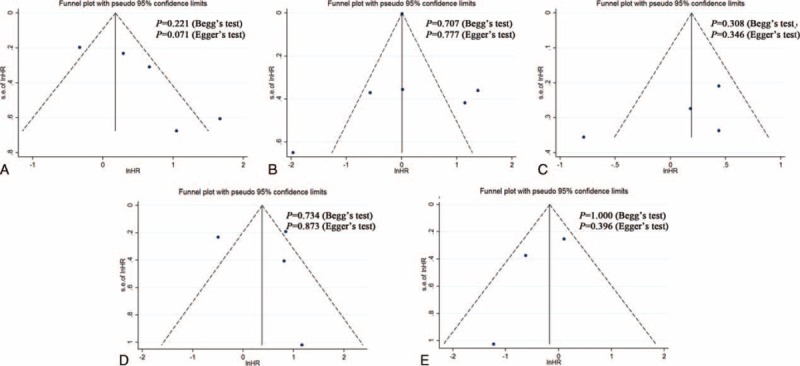
Funnel plots, Begg and Egger’ test results for the evaluation of publication bias. (A) HIF-1α and OS, (B) HIF-1α and CSS, (C) HIF-1α and PFS, (D) HIF-2α and CSS, (E) HIF-2α and PFS. CSS = cancer-specific survival, HIF = hypoxia-inducible factor, HR = hazard ratio, OS = overall survival, PFS = progression-free survival.

The publication bias of our meta-analysis was assessed using funnel plots and Begg's and Egger's tests. As shown in Figure 4, no evidence of significant publication bias was found.

## DISCUSSION

HIF isoforms, which are optimally characterized as markers mediating cellular adaptation to hypoxic stress,^31,32^ have long been associated with alterations to vessel function by transcriptionally activating specific HIF-responsive genes.^33^ Under normoxia, HIF is targeted and ubiquitinated by pVHL, leading to subsequent degradation by cellular proteasome. However, given that VHL inactivation was reported in ∼80% of sporadic ccRCC,^6^ loss of functional pVHL impairs the destabilization of HIF isoforms even in normoxic conditions. Therefore, inappropriate activation of downstream target genes that would normally only be activated in hypoxic conditions were promoted, contributing to ccRCC development.^7^ Given that the hypoxia-inducible pathway plays a critical role in ccRCC, and clear cell histology represents the most common subtype of RCC, numerous studies were performed to assess the prognostic values of HIF for RCC patients. Nevertheless, inconsistent reports generated inconclusive results. In this regard, we conducted a meta-analysis of all eligible studies to provide a systematic review of HIF expression on the prognosis of RCC patients.

In the present study, we evaluated the prognostic values of HIF-1α and HIF-2α independently because of their distinct target genes and potentially nonoverlapping properties. When initially analyzed as a whole, no significant association was identified between HIF-1α expression and OS (HR 1.637, 95% CI 0.898–2.985, *P* = 0.108), CSS (HR 1.110, 95% CI 0.595–2.069, *P* = 0.744), and PFS (HR 1.113, 95% CI 0.675–1.836, *P* = 0.674), although patients with high HIF-1α level tended to show a relatively unfavorable prognosis. Similar insignificant results were obtained for HIF-2α between high and low expression in terms of CSS (HR 1.597, 95% CI 0.667–3.824, *P* = 0.293) and PFS (HR 0.847, 95% CI 0.566–1.266, *P* = 0.417).

Subgroup analysis was performed to explain the heterogeneity of pooled HRs and reveal detailed information of the HIF function. Considering that recent evidence indicated that different subcellular localizations of HIF expression might exert different effects on RCC^21,24^ and the same mechanism was shown in other proteins, such as p21,^34^ we performed subgroup analysis by first stratifying studies as the nuclear expression group or cytoplasmic expression group. The high nuclear expression of HIF-1α was discovered to be significantly associated with poor OS (HR 2.014, 95% CI 1.206–3.363, *P* = 0.007), whereas patients with high cytoplasmic expression of HIF-1α tended to demonstrate a more favorable prognosis in terms of OS, though not reaching a statistical significance (HR 0.720, 95% CI 0.490–1.059, *P* = 0.095). The same conflicting trend was also observed in the subgroup analyses of the effects of nuclear and cytoplasmic HIF-1α expression on CSS, although the difference was not significant. HIF-1α was considered an unfavorable prognostic marker in various cancers,^15,16,35^ but the underlying mechanism of the opposing effects of HIF-1α overexpression in different subcellular compartments remains speculative. A possible explanation indicates that HIF-1α is a nuclear transcription factor that is functional only in the nucleus, and a high cytoplasmic expression of HIF-1α suggests that this factor has translocated to the cytoplasm and thus unable to activate HIF-responsive genes, leading to a relatively good prognosis.^21^ For HIF-2α, subgroup analysis revealed that high cytoplasmic expression of HIF-2α was significantly associated with poor CSS (HR 2.356, 95% CI 1.629–3.407, *P* = 0.000). Accumulating evidence demonstrates that HIF-2α, rather than HIF-1α, is the primary oncogenic driver in RCC and can act as a nuclear tumor promoter.^36,37^ However, unlike HIF-1α, HIF-2α plays a unique role in the tumor cytoplasm. Uniacke et al^38^ demonstrated that HIF-2α can form a complex in the cytoplasm to help initiate protein synthesis in periods of oxygen scarcity and eukaryotic translation initiation factor 4E (eIF4E) inhibition. This mechanism concerning HIF-2α function in the cytoplasm is very important for tumor development as it evaded hypoxia-induced repression of protein translation, which can explain the association between HIF-2α with high cytoplasmic expression and unfavorable prognosis in RCC patients.

Given that the HIF expression is associated with the alteration in the VEGF pathway, the HIF expression levels may provide therapeutic implications in the selection of patients who will benefit from new targeted antiangiogenic therapies. Unfortunately, our subgroup analyses for patients who received tyrosine kinase inhibitors (Sunitinib or Pazopanib) did not show any significant benefit for survival outcomes in terms of different levels of HIFs, even though several single studies reached significant statistics with opposing results.^22,23,30^ This discrepancy and insignificant pooled results may be attributed to limited studies and different follow-up time in each study. Thus, future investigations with large cohort and sufficient follow-up are warranted to reach a consensus on patient selection for targeted therapy based on the HIF expression.

The present study comprehensively evaluated the effect of HIFs on the prognosis of RCC patients, but several limitations should be noted. First, although IHC is the standard method to evaluate the HIF expression, the cutoff value for high or low levels of HIFs varied in different studies, which might cause heterogeneity of the overall results. Therefore, a more unanimous cutoff value for the definition of high HIF expression is recommended for future studies. Second, a remarkable heterogeneity was observed in certain categories of analysis. Although the heterogeneity was attributed by subgroup analysis to the subcellular location of HIF expression, this basis cannot cover the entire source. Third, several studies included in the analysis did not directly provide HRs and should be calculated using the methods recommended by Tierney et al,^18^ which somehow rendered the extracted data less precise. Fourth, relatively few studies were included in this meta-analysis and it could lead to a premature result. So it may need an update in the future when more eligible studies are published. Finally, as a literature-based analysis, the fact that studies with positive results were more likely to be published can amplify the association between HIF expression and survival outcomes, which can lead to publication bias,^39^ although this bias was not detected in the current analysis.

In conclusion, this meta-analysis is the first to evaluate the influence of the expression of different HIF isoforms on the prognosis of RCC patients. This study showed that high nuclear expression of HIF-1α and high cytoplasmic expression of HIF-2α indicate unfavorable prognosis in patients with RCC, which may potentially serve as risk stratification markers and even therapeutic targets to manage this disease.
